# Correction: Thorax temperature and niche characteristics as predictors of abundance of Amazonian Odonata

**DOI:** 10.1371/journal.pone.0342615

**Published:** 2026-02-12

**Authors:** PLOS One

## Notice of Republication

This article was republished on January 27, 2026, to correct the incorrect figures; these errors were introduced during typesetting.

Professor “Alex Córdoba-Aguilar” should be listed instead of “Alex Córdoba-Aguillar.” We also corrected the missing funding BRC number, 076.

## Supporting information

S1 File Originally published, uncorrected article. (PDF)

S2 File Republished, corrected article. (PDF)
