# Supplementary material for: Thorax temperature and niche characteristics as predictors of abundance of Amazonian Odonata
Source: PLoS One. 2025 Jun 26;20(6):e0311072. doi: 10.1371/journal.pone.0311072 (PMC12200694; doi:10.1371/journal.pone.0311072)
Supplement: Table S1 — (DOCX) [file pone.0311072.s001.docx]

**Table S1.** Environmental information of the 46 streams sampled in the Northeastern and Southeastern of Pará, Brazil.

| **Streams** | **City** | **Longitude** | **Latitude** | **Mesoregion** | **Land use** | **Width** | **Depth** | **HII** |
| --- | --- | --- | --- | --- | --- | --- | --- | --- |
| ua1 | Parauapebas | -589387 | -9318186 | Southeastern | Mining | 1.30 | 75.00 | 0.93 |
| ua2 | Parauapebas | -592498 | -9316489 | Southeastern | Mining | 1.63 | 27.43 | 0.93 |
| ua3 | Canaã dos Carajás e Parauapebas | -596329 | -9315144 | Southeastern | Mining | 1.47 | 56.67 | 0.93 |
| ua4 | Parauapebas | -591097 | -9323315 | Southeastern | Mining | 1.50 | 34.00 | 0.93 |
| ua5 | Canaã dos Carajás | -565204 | -9300425 | Southeastern | Mining | 4.17 | 27.33 | 0.93 |
| ua6 | Canaã dos Carajás | -562258 | -9299966 | Southeastern | Mining | 4.63 | 11.67 | 0.90 |
| ua7 | Canaã dos Carajás e Parauapebas | -563567 | -9297398 | Southeastern | Mining | 1.37 | 20.67 | 0.93 |
| ua8 | Canaã dos Carajás | -561932 | -9296038 | Southeastern | Mining | 0.93 | 20.67 | 0.95 |
| ua9 | Canaã dos Carajás e Parauapebas | -574382 | -9325723 | Southeastern | Mining | 0.93 | 19.72 | 0.57 |
| ua10 | Parauapebas | -576619 | -9322904 | Southeastern | Mining | 1.20 | 27.43 | 0.92 |
| ua11 | Parauapebas | -596329 | -9315144 | Southeastern | Mining | 0.65 | 12.00 | 0.76 |
| ua12 | Canaã dos Carajás e Parauapebas | -591097 | -9323315 | Southeastern | Mining | 0.93 | 19.72 | 0.78 |
| ua13 | Canaão dos Carajás e Parauapebas | -588232 | -9325877 | Southeastern | Mining | 4.93 | 11.67 | 0.90 |
| ua14 | Parauapebas | -591141 | -9326591 | Southeastern | Mining | 1.20 | 83.33 | 0.94 |
| ua15 | Canaã dos Carajás e Parauapebas | -588588 | -9328251 | Southeastern | Mining | 3.33 | 20.33 | 0.90 |
| ua16 | Parauapebas | -595239 | -9325612 | Southeastern | Mining | 3.27 | 21.67 | 0.92 |
| ua17 | Parauapebas | -595823 | -9324955 | Southeastern | Mining | 4.27 | 20.67 | 0.86 |
| ua18 | Canaã dos Carajás e Parauapebas | -598084 | -9326759 | Southeastern | Mining | 0.67 | 33.33 | 0.90 |
| ua19 | Parauapebas | -598260 | -9327085 | Southeastern | Mining | 1.07 | 73.33 | 0.90 |
| ua20 | Canaã dos Carajás | -575618 | -9293747 | Southeastern | Mining | 2.30 | 19.17 | 0.88 |
| ua21 | Canaã dos Carajás | -573026 | -9295313 | Southeastern | Mining | 5.17 | 29.67 | 0.91 |
| ua22 | Canaã dos Carajás | -569778 | -9294768 | Southeastern | Mining | 6.33 | 15.33 | 0.91 |
| ua23 | Canaã dos Carajás e Parauapebas | -561932 | -9296038 | Southeastern | Mining | 4.60 | 21.39 | 0.95 |
| ua24 | Paragominas | -47.86841 | -3.25637 | Southeastern | Multiples | 4.98 | 41.60 | 0.56 |
| ua25 | Paragominas | -47.781667 | -3.206861 | Southeastern | Multiples | 5.26 | 45.71 | 0.50 |
| ua26 | Paragominas | -47.775694 | -3.253583 | Southeastern | Multiples | 1.71 | 23.84 | 0.55 |
| ua27 | Paragominas | -47.766167 | -3.276778 | Southeastern | Multiples | 2.67 | 31.20 | 0.54 |
| ua28 | Paragominas | -47.752333 | -3.225194 | Southeastern | Multiples | 4.23 | 48.22 | 0.55 |
| ua29 | Paragominas | -47.72015 | -3.24875 | Southeastern | Multiples | 2.45 | 39.20 | 0.48 |
| ua30 | Paragominas | -47.760778 | -3.258139 | Southeastern | Multiples | 3.01 | 29.49 | 0.53 |
| ua31 | Paragominas | -47.746583 | -3.204389 | Southeastern | Multiples | 3.83 | 30.50 | 0.43 |
| ua32 | Paragominas | -47.737861 | -3.233583 | Southeastern | Multiples | 2.21 | 11.42 | 0.48 |
| ua33 | Paragominas | -47.72747 | -3.32948 | Southeastern | Multiples | 1.87 | 15.67 | 0.55 |
| ua34 | Paragominas | -47.67822 | -3.20925 | Southeastern | Multiples | 4.14 | 46.35 | 0.39 |
| ua35 | Paragominas | -47.66307 | -3.28298 | Southeastern | Multiples | 2.38 | 26.18 | 0.71 |
| ua36 | Paragominas | -47.66903 | -3.29055 | Southeastern | Multiples | 3.34 | 39.44 | 0.59 |
| ua37 | Paragominas | -47.663111 | -3.304861 | Southeastern | Multiples | 2.49 | 15.68 | 0.61 |
| ua38 | Paragominas | -47.70055 | -3.17011 | Southeastern | Multiples | 2.42 | 14.89 | 0.52 |
| ua39 | Paragominas | -47.62288 | -3.27841 | Southeastern | Multiples | 3.36 | 40.80 | 0.59 |
| ua40 | Ipixuna do Pará | -47.52926 | -2.55115 | Northeastern | Pasture | 4.75 | 35.13 | 0.57 |
| ua41 | Aurora do Pará | -47.563372 | -2.133405 | Northeastern | Urban area | 2.31 | 29.09 | 0.07 |
| ua42 | Concórdia do Pará | -47.9519 | -1.99732 | Northeastern | Urban area | 1.89 | 11.44 | 0.22 |
| ua43 | Concórdia do Pará | -48.03743 | -1.98979 | Northeastern | Urban area | 2.27 | 16.18 | 0.64 |
| ua44 | Tomé-Açú | -48.14775 | -2.4085 | Northeastern | Urban area | 3.16 | 26.65 | 0.33 |
| ua45 | Tomé-Açú | -48.228305 | -2.394137 | Northeastern | Urban area | 3.92 | 44.04 | 0.61 |
| ua46 | Tomé-Açú | -48.25435 | -2.43181 | Northeastern | Urban area | 1.85 | 9.22 | 0.51 |

Multiples (secondary forest, agriculture, pasture and bauxite mining); HII (Habitat Integrity Index).
