# Supplementary material for: Thorax temperature and niche characteristics as predictors of abundance of Amazonian Odonata
Source: PLoS One. 2025 Jun 26;20(6):e0311072. doi: 10.1371/journal.pone.0311072 (PMC12200694; doi:10.1371/journal.pone.0311072)
Supplement: Table S2 — (DOCX) [file pone.0311072.s002.docx]

**Table S2.** Odonata species. SD stands for standard deviation.

| **Family/Species/Author** | **Code** | **Thorax width (cm)** | **SD** | **Thorax temperature (°C)** | **SD** | **Air Temperature** | **SD** | **Abundance** |
| --- | --- | --- | --- | --- | --- | --- | --- | --- |
| **COENAGRIONIDAE** |  |  |  |  |  |  |  |  |
| *Acanthagrion kennedii* Williamson, 1916 | *A kennedii* | 1.281 | 0.187 | 29.533 | 2.981 | 31.733 | 1.294 | 8 |
| *Acanthallagma luteum* Williamson & Williamson, 1924 | *A luteum* | 1.390 | NA | 29.800 | NA | 29.900 | NA | 1 |
| *Argia collata* Selys, 1865 | *A collata* | 1.512 | 0.151 | 29.879 | 2.284 | 30.063 | 1.62 | 47 |
| *Argia dives* Förster, 1914 | *A dives* | 2.310 | 0.396 | 35.800 | 0.707 | 33.000 | 0.424 | 3 |
| *Argia fumigata* Hagen in Selys, 1865 | *A fumigata* | 1.760 | NA | 27.300 | NA | 26.800 | NA | 4 |
| *Argia indicatrix* Calvert, 1902 | *A indicatrix* | 1.311 | 0.147 | 30.004 | 1.862 | 30.409 | 1.758 | 36 |
| *Argia infumata* Selys, 1865 | *A infumata* | 1.397 | 0.223 | 30.950 | 2.057 | 30.175 | 1.615 | 4 |
| *Argia oculata* Hagen in Selys, 1865 | *A oculata* | 1.996 | 0.165 | 30.860 | 1.616 | 30.600 | 1.768 | 31 |
| *Argia reclusa* Selys, 1865 | *A reclusa* | 1.710 | NA | 27.600 | NA | 28.100 | NA | 1 |
| *Argia tinctipennis* Selys, 1865 | *A tinctipennis* | 1.560 | 0.216 | 30.037 | 2.065 | 30.133 | 1.777 | 38 |
| *Epipleoneura kaxuriana* Machado, 1985 | *E kaxuriana* | 1.035 | 0.134 | 28.550 | 0.778 | 28.500 | 3.111 | 3 |
| *Epipleoneura metallica* Rácenis, 1955 | *E metallica* | 0.983 | 0.205 | 27.744 | 2.538 | 29.009 | 2.043 | 62 |
| *Metaleptobasis bicornis* (Selys, 1877) | *M bicornis* | 1.788 | 0.049 | 28.900 | 1.807 | 28.200 | 0 | 4 |
| *Neoneura luzmarina* De Marmels, 1989 | *N luzmarina* | 1.454 | 0.159 | 29.276 | 1.925 | 30.706 | 1.913 | 30 |
| *Phasmoneura exigua* (Selys, 1886) | *P exigua* | 1.253 | 0.118 | 29.500 | 2.261 | 29.967 | 2.757 | 3 |
| *Phoenicagrion flammeum* (Selys, 1876) | *P flammeum* | 1.550 | NA | 30.800 | NA | 30.900 | NA | 1 |
| *Protoneura tenuis* Selys, 1860 | *P tenuis* | 1.126 | 0.094 | 27.212 | 1.461 | 29.205 | 1.121 | 17 |
| *Psaironeura tenuissima* (Selys, 1886) | *P tenuissima* | 0.946 | 0.116 | 28.900 | 1.552 | 30.500 | 2.395 | 15 |
| *Telebasis griffinii* (Martin, 1896) | *T griffinii* | 1.690 | NA | 27.500 | NA | 27.600 | NA | 1 |
| *Tigriagrion aurantinigrum* Calvert, 1909 | *T aurantinigrum* | 1.010 | NA | 30.000 | NA | 32.200 | NA | 1 |
| **CALOPTERYGIDAE** |  |  |  |  |  |  |  |  |
| *Hetaerina amazonica* Sjöstedt, 1918 | *H amazonica* | 2.390 | NA | 29.300 | NA | 27.700 | NA | 1 |
| *Hetaerina indeprensa* Garrison, 1990 | *H indeprensa* | 2.671 | 0.273 | 29.745 | 1.472 | 29.945 | 1.559 | 52 |
| *Hetaerina rosea* Selys, 1853 | *H rosea* | 2.485 | 0.205 | 30.650 | 1.768 | 31.750 | 1.061 | 2 |
| *Mnesarete aenea* (Selys, 1853) | *M aenea* | 2.062 | 0.230 | 29.495 | 1.960 | 29.585 | 1.354 | 92 |
| *Mnesarete cupraea* (Selys, 1853) | *M cupraea* | 2.431 | 0.150 | 28.200 | 2.261 | 30.180 | 0.882 | 20 |
| *Mnesarete williamsoni* Garrison, 2006 | *M williamsoni* | 2.098 | 0.141 | 29.133 | 1.288 | 29.744 | 1.415 | 5 |
| **HETERAGRIONIDAE** |  |  |  |  |  |  |  |  |
| *Heteragrion bariai* De Marmels, 1989 | *H bariai* | 1.748 | 0.236 | 29.300 | 0.623 | 30.150 | 2.725 | 6 |
| *Heteragrion icterops* Selys, 1862 | *H icterops* | 1.620 | NA | 27.300 | NA | 31.200 | NA | 1 |
| *Heteragrion silvarum* Sjöstedt, 1918 | *H silvarum* | 1.827 | 0.254 | 29.011 | 1.469 | 28.911 | 2.002 | 28 |
| *Oxystigma petiolatum* (Selys, 1862) | *O petiolatum* | 1.585 | 0.058 | 27.650 | 0.998 | 29.225 | 1.837 | 4 |
| **POLYTHORIDAE** |  |  |  |  |  |  |  |  |
| *Chalcopteryx rutilans* (Rambur, 1842) | *C rutilans* | 1.543 | 0.208 | 30.027 | 1.619 | 30.420 | 1.715 | 156 |
| **PERILESTIDAE** |  |  |  |  |  |  |  |  |
| *Perilestes solutus* Williamson & Williamson, 1924 | *P solutus* | 1.710 | NA | 28.400 | NA | 30.600 | NA | 1 |
| **LIBELLULIDAE** |  |  |  |  |  |  |  |  |
| *Anatya guttata* (Erichson in Schomburgk, 1848) | *A guttata* | 2.960 | NA | 28.900 | NA | 29.300 | NA | 1 |
| *Brachymesia herbida* (Gundlach, 1889) | *B herbida* | 5.020 | NA | 40.400 | NA | 35.400 | NA | 1 |
| *Dasythemis esmeralda* Ris, 1910 | *D esmeralda* | 3.270 | 0.235 | 31.300 | 0.529 | 27.933 | 0.473 | 4 |
| *Diastatops obscura* (Fabricius, 1775) | *D obscura* | 4.310 | 0.156 | 33.725 | 2.320 | 32.175 | 0.275 | 4 |
| *Elasmothemis cannacrioides* (Calvert, 1906) | *E cannacrioides* | 4.750 | NA | 38.000 | NA | 33.500 | NA | 1 |
| *Erythrodiplax basalis* (Kirby, 1897) | *E basalis* | 2.686 | 0.324 | 32.913 | 3.610 | 31.595 | 2.951 | 36 |
| *Erythrodiplax castanea* (Burmeister, 1839) | *E castanea* | 3.490 | NA | 38.800 | NA | 30.200 | NA | 1 |
| *Erythrodiplax fusca* (Rambur, 1842) | *E fusca* | 2.861 | 0.455 | 35.647 | 4.143 | 32.437 | 3.790 | 16 |
| *Fylgia amazonica* Kirby, 1889 | *F amazonica* | 2.006 | 0.188 | 28.785 | 2.137 | 29.336 | 1.483 | 43 |
| *Micrathyria pseudeximia* Westfall, 1992 | *M pseudeximia* | 2.385 | 0.431 | 25.100 | 0.000 | 31.200 | 0.000 | 1 |
| *Nephepeltia phryne* (Perty, 1833) | *N phryne* | 2.350 | NA | 31.300 | NA | 32.600 | NA | 1 |
| *Oligoclada abbreviata* (Rambur, 1842) | *O abbreviata* | 2.722 | 0.197 | 34.048 | 1.844 | 31.186 | 1.581 | 15 |
| *Oligoclada walkeri* Geijskes, 1931 | *O walkeri* | 2.586 | 0.378 | 33.850 | 2.655 | 29.762 | 2.163 | 9 |
| *Orthemis* sp.1 | *Orthemis sp1* | 6.532 | 0.605 | 36.100 | 4.332 | 29.940 | 3.625 | 5 |
| *Perithemis electra* Ris, 1930 | *P electra* | 2.598 | 0.179 | 32.460 | 3.611 | 29.860 | 1.284 | 1 |
| *Perithemis lais* (Perty, 1833) | *P lais* | 2.031 | 0.257 | 32.383 | 1.877 | 31.331 | 3.000 | 21 |
| *Perithemis mooma* Kirby, 1889 | *P mooma* | 2.310 | 0.057 | 34.350 | 1.344 | 36.600 | 2.121 | 6 |
| *Perithemis thais* Kirby, 1889 | *P thais* | 1.994 | 0.194 | 31.043 | 2.380 | 30.557 | 1.667 | 7 |
| *Rhodopygia cardinalis* (Erichson in Schomburgk, 1848) | *R cardinalis* | 4.510 | NA | 38.200 | NA | 32.600 | NA | 1 |
| *Uraris fastigiata*  (Burmeister, 1839) | *U fastigiata* | 3.740 | NA | 36.600 | NA | 34.000 | NA | 1 |
| *Zenithoptera lanei* Santos, 1941 | *Z lanei* | 2.730 | NA | 36.800 | NA | 32.700 | NA | 1 |
| **GOMPHIDAE** |  |  |  |  |  |  |  |  |
| *Zonophora batesi* Selys, 1869 | *Z batesi* | 7.510 | 0.424 | 34.900 | 0.424 | 31.300 | 0.000 | 2 |
