# Supplementary material for: Thorax temperature and niche characteristics as predictors of abundance of Amazonian Odonata
Source: PLoS One. 2025 Jun 26;20(6):e0311072. doi: 10.1371/journal.pone.0311072 (PMC12200694; doi:10.1371/journal.pone.0311072)
Supplement: Table S3 — (DOCX) [file pone.0311072.s003.docx]

**Table S3.** Odonata that are only collected in streams with higher (>0.7) and low (0.7) habitat integrity index (HII).

| **Streams with low HII** | | **Streams with greater HII** | |
| --- | --- | --- | --- |
| **Zygoptera** | **Anisoptera** | **Anisoptera** | **Zygoptera** |
| *A kennedii* | *B herbida* | *A guttata* | *A dives* |
| *A luteum* | *D obscura* | *U fastigiata* | *A fumigata* |
| *A infumata* | *E cannacrioides* |  | *A oculata* |
| *A reclusa* | *E basalis* |  | *H amazonica* |
| *E kaxuriana* | *E castanea* |  | *H bariai* |
| *H icterops* | *E fusca* |  | *Het.indeprensa* |
| *Het.rosea* | *F amazonica* |  | *Met.bicornis* |
| *M williamsoni* | *M pseudeximia* |  |  |
| *O petiolatum* | *N luzmarina* |  |  |
| *P solutus* | *N phryne* |  |  |
| *P flammeum* | *O abbreviata* |  |  |
| *T griffinii* | *Orthemis sp1* |  |  |
| *T aurantinigrum* | *P electra* |  |  |
|  | *P lais* |  |  |
|  | *P mooma* |  |  |
|  | *P thais* |  |  |
|  | *R cardinalis* |  |  |
|  | *Z lanei* |  |  |
