# Supplementary material for: Thorax temperature and niche characteristics as predictors of abundance of Amazonian Odonata
Source: PLoS One. 2025 Jun 26;20(6):e0311072. doi: 10.1371/journal.pone.0311072 (PMC12200694; doi:10.1371/journal.pone.0311072)
Supplement: Attachment — Submitted filename: Response to Reviewers (2).docx [file pone.0311072.s004.docx]

Editor

[Comment 1] After this new review round, unfortunately, new reviewers needed to revise the text since the original ones were not available. One of the reviewers decided for minor reviews for your manuscript in this new submission. Still, the other new reviewer indicated major reviews are still necessary to make your manuscript scientifically meaningful. Therefore, considering the proposed improvements, I believe your manuscript still needs a (new) major review before it is accepted for publication. Considering the amount of change and the time of the year, I will grant you four months to prepare the new version of your manuscript. Therefore, please resubmit a new version of your manuscript by March, 15 2024, along with a rebuttal letter informing about all the changes that were and were not made, explaining to the reviewers why this or that suggestion was not performed.

**Author’s Response:** Thank you for the valuable feedback provided by the new set of reviewers. I acknowledge the importance of addressing these concerns thoroughly, and I am committed to implementing the necessary changes to improve the overall quality of the manuscript. We leave the changes highlighted in red in change control. I will ensure that the proposed improvements are incorporated effectively to meet the standards of PLOS ONE.

Reviewer #3:

[Comment 2] This manuscript has interesting data within it, and the field work has obviously involved significant work. However, there are a number of issues within the paper that need resolving. In particular, I recommend that the paper is thoroughly checked for its grammar as there are a number of places where careful editing is needed to ensure it makes sense. Additionally, there are some potential issues with the methods which need further explanation to convey confidence in the methodology. Examples of problems are as below

**Author’s Response:** I appreciate your recognition of the interesting data and the significant effort invested in the fieldwork. I will thoroughly address the grammar issues and ensure careful editing for clarity. Additionally, I will provide further explanation in the methods section to enhance confidence in the methodology. Your input is invaluable, and I am committed to resolving these concerns in the revised manuscript.

[Comment 3] Line 54/56. This is not a logical sentence and leads to confusion about what is meant. Do you mean species richness or individual species abundances? Logic dictates that a wide range of resource types would increase richness, but individual abundances would be governed by individual species resource abundances.

**Author’s Response:** Thank you for the comment, in this article we are talking about individual abundances. We tried to modify the paragraph here.

Line 40-44: The hypothesis that links niche breadth/tolerance, predicts that populations that can remain viable in a wide range of environmental conditions have greater abundance. In this context, they can be characterized as generalists (occur in a wide range of environmental conditions) and specialists (occur in a more restricted range).While the hypothesis for the niche position predicts that non-marginal species, those that occur in a larger availability of habitats, tend to be more abundant(11).

[Comment 4] Line 56-57 Again it is unclear what is meant here – Reference to https://doi.org/10.1111/geb.13139 might clarify this section on niche breadth and position.

**Author’s Response:** Thank you for the comment and reference. We tried to modify the paragraph here.

Line 40-44: The hypothesis that links niche breadth/tolerance, predicts that populations that can remain viable in a wide range of environmental conditions have greater abundance. In this context, they can be characterized as generalists (occur in a wide range of environmental conditions) and specialists (occur in a more restricted range).While the hypothesis for the niche position predicts that non-marginal species, those that occur in a larger availability of habitats, tend to be more abundant(11).

[Comment 5] Line 62 -replace ‘than what occurs with specialist species’ with ‘than the contribution of specialist species’

**Author’s Response:** Done.

[Comment 6] Line 76 do you need reference to canopy loss here?

**Author’s Response:** Following the reviewer's suggestion, we have included the reference to Castillo-Pérez et al. (2020), which addresses the “Insect thermal limits in warm and perturbed habitats: Dragonflies and damselflies as study cases”.

[Comment 7] Line 82/83 change end of sentence to ‘thereby altering odonate community composition’

**Author’s Response:** Thank you for the comment We tried to modify some paragraphs and lot of sentences was removed.

*.*

[Comment 8] Line 90-92 Not a sentence

**Author’s Response:** I did not understand.

[Comment 9] Line 118 For the non-specialist explain what 1st to 3rd order streams are

**Author’s Response:** Line 100:101: It means streams with an average width of 2.8 meters and a depth of 29.9 centimeters. We chose to include this information in the manuscript rather than describing the entire Strahler classification system, which might not be clear to the reader.

[Comment 10] Insect sampling- explain how many visits were made to each 150m length sampling unit and was sam-ping intensity the same at all sites.

**Author’s Response:** Only one visit was made to each stream, and the sampling intensity was consistent across all locations. These details have been included in the manuscript. Line 102-104: Adult odonates were collected only once stream, in 20 segments spaced 5m apart, distributed along a continuous 150-meter longitudinal stretch in each stream [further details in 20, 27, 28]*.*

[Comment 11] For the environmental variables, were these collected for each of the 20m segments of each stream sampled and if so how was variation in the environmental parameters taken into account in the analysis.

**Author’s Response:** It doesn't take it into consideration, after all, the data is combined and the stream becomes a sample, or in the case of this article, the species are samples. The segments is a pseudoreplication. But in the field we proceed like this, with these short spacings, to make it easier to cover the habitats of all species.

[Comment 12] Line 150 to 154. Whilst HII is used widely, more explanation of the precise measures taken for each variable is needed to help the reader.

**Author’s Response:** Here we rewrite metrics name to facilitate this understanding.

Line 130-134: The HII was composed by 12 items that describe the degree of habitat integrity in the stream: land use pattern beyond the riparian zone; width of riparian forest; **c**ompleteness of riparian forest, **v**egetation of riparian zone within 10 m of channel,; retention devices and sediment in the channel; river bank structure; **b**ank undercutting, **s**tream bottom,; distribution of riffles and pools; characteristics of aquatic vegetation and **d**etritus [37].

[Comment 13] In line 151 is width the width of the riparian zone? If it is stream width then including width as an important descriptor of odonate composition would not be valid in line 241 if it is within the HII index.

**Author’s Response:** Thank you very much for the comment. They are different measurements, but we rewrite metrics name to avoid this confusion.

Line 127: For each stream, four environmental variables were measured: depth (cm), channel width (m)…

Line 130-134: The HII was composed by 12 items that describe the degree of habitat integrity in the stream: land use pattern beyond the riparian zone; width of riparian forest; **c**ompleteness of riparian forest, **v**egetation of riparian zone within 10 m of channel,; retention devices and sediment in the channel; river bank structure; **b**ank undercutting, **s**tream bottom,; distribution of riffles and pools; characteristics of aquatic vegetation and **d**etritus [37].

[Comment 14] Air temperature was measured with a Testo 805 infrared thermometer. This takes spot readings of surface temperature so it is not obvious how this can be used to measure air temperature; explanation is needed. Would it not be measuring the surface temperature of the vegetation, which is not going to be the same as air temperature?

**Author’s Response:** We pointed the thermometer towards where the insect was sitting, as the thermometer does not have the range to measure very far away, we believe it was successful in measuring the temperature of the air and the microhabitat in which it is located.

[Comment 15] With the use of averages in the OMI analysis is the detail of the environmental spaces occupied by species loosing detail?

**Author’s Response:**: we did not understand this.

[Comment 16] Results – text needs checking for sense and use of English language, e.g. line 205 temperature differences should be expressed as ‘greater’, not ‘higher’ and line 214 should read ‘Both thoracic width and air temperature affect thoracic temperature’. Line 223 to 226. If ranges of thoracic temperatures are compared to air temperatures then ranges need to be expressed for both Anisoptera and Zygoptera. Line 225 ,rewrite as between 4 degrees below and 2 degrees above. If air temperature is the ambient temperature then how can thoracic temperature be below ambient? Does it not imply that the air temperature measured at the location of capture was not the same as that where the individual equilibrated its body temperature? Does this make the results invalid?

**Author’s Response:** Thank you, we corrected the English*.* We pointed the thermometer towards where the insect was sitting, as the thermometer does not have the range to measure very far away, we believe it was successful in measuring the temperature of the air and the microhabitat in which it is located.

[Comment 17] Figure 4 is useful and illustrates the point being made well, but it would be much more informative if range bars were given for the individual species (where available).

**Author’s Response:** Done. We had to separate the suborders to be more organized. However, we should add at manuscript also?

[Comment 18] Line 269 ‘When combined niche position was important for Odonata SCBD’, not ‘separated’ as written.

**Author’s Response:** Thank you done.

[Comment 19] The information within the discussion is there but a thorough rewrite is probably needed, especially in relation to the use of English, and occasional logical inconsistencies. For example:

**Author’s Response:** Thank you. We agree with the notes, and therefore, we have rewritten a lot of topics of discussion.

[Comment 20] Line 306-308 needs completely rewriting- it does not make sense.

**Author’s Response:** Thank you. We agree with the notes, and therefore, we have rewritten a lot of topics of discussion.

[Comment 21] Line 320 – the study is on adult odonates so reference to body temperatures in relation to water depth is not logical.

**Author’s Response:** Thank you is a mistake. We agree with the notes, and therefore, we have rewritten a lot of topics of discussion.

[Comment 22] Line 325 Thoracic width, not chest width

**Author’s Response:** Thank you done.

[Comment 23] Line 328 – see previous comments above about comparing measurements of body temperatures and air temperatures and the actual method of measuring air temperature.

**Author’s Response:** Thank you, we corrected the English. We pointed the thermometer towards where the insect was sitting, as the thermometer does not have the range to measure very far away, we believe it was successful in measuring the temperature of the air and the microhabitat in which it is located.

[Comment 24] Line 332 whilst small bodied species may loose heat by convection, body temperature can still not go below air temperature.

**Author’s Response:** Yes, it may not go down, but it may be very close to the air temperature, so we hope to detect those that heat by convection.

[Comment 25] Lines 336-340 are not needed.

**Author’s Response:** Thank you, we remove*.*

[Comment 26] The last two sentences of the discussion do not provide a good ending to the manuscript. Something along the lines of ‘We have demonstrated that adult odonate species composition varies in relation to air temperature, because of the different relationships of the different orders to air temperature and to aspects of riparian disturbance. We therefore suggest that their monitoring would provide a good indicator of riparian quality.’

**Author’s Response:** Thank you so Much. Finally, niche characteristics may be important for the distribution of Odonata. Line 297-304: ecophysiological traits also was important for Anisoptera and Zygoptera abundance. May [15] suggests also that climate, body size and behavior are essential for maintaining the body temperature of Odonata**.** Changes in streams due of anthropic activities alter microclimatic patterns such as air temperature, fundamental for the physiological processes of Odonata species, leading to a change in the composition of species in these environments [38]. We have demonstrated that adult odonate species composition varies in relation to habitat integrity. We therefore suggest that their monitoring would provide a good indicator of riparian zone quality considering niche characteristics and their thermorregulation habilities.

Reviewer #4:

[Comment 27] I would like to commend the innovative and intriguing nature of your research. The sampling, data analysis, and methodology employed are exemplary and demonstrate a high level of rigor. However, upon reviewing the manuscript, I have observed that the discussion section requires improvement before it can be considered ready for publishing. I have provided comments and suggestions within the manuscript, focusing on the most critical areas that need further development. Additionally, I've outlined a few minor corrections that require attention. Your work holds significant promise, and with refinements in the discussion section, I am confident it will meet the high standards set by the rest of the research. I eagerly anticipate the enhancements and believe that once refined, your work will be well-prepared for publication, potentially making a meaningful contribution to the field.

**Author’s Response:** Thank you so much.

[Comment 28] L28: was

**Author’s Response:** Done.

[Comment 29] L 57: Reference should be in numerical order [2, 7-8]. This should be amended in all the following cases. In the case of 48-49 they are referenced later than 50, so this should be amended.

**Author’s Response:** We conducted a review of the entire manuscript to eliminate chronological inconsistencies in the references. We emphasize that the references used after 50 revisit sources that are crucial for discussing the results. We included the initial chronology of when they were cited in the text, ensuring no duplication of citations*.*

[Comment 30] L 67: exclude (Insecta: Odonata; two suborders; Anisoptera, and Zygoptera)

**Author’s Response:** Done.

[Comment 31] L68: exclude ‘e.g..”

**Author’s Response:** Done.

[Comment 32] L70: Castillo-Pérez [12]

**Author’s Response:** Done.

[Comment 33] L73: it should be "thermal conformers or heliotherms"

**Author’s Response:** We agree with the reviewer's suggestion and have made the modifications accordingly.

[Comment 34] L76: species

**Author’s Response:** Done.

[Comment 35] L82-83: acting as an environmental.

**Author’s Response:** We conducted a thorough review of the manuscript and incorporated the reviewer's suggestion into the introduction.

[Comment 36] L 118: Strahler [26]

**Author’s Response:** Done.

[Comment 37] L 135: Why are you using only males?

**Author’s Response:** We used only male individuals because species identification is possible in this sex. These details have been included in the manuscript.

[Comment 38] L143: than one

**Author’s Response:** We rewrote the sentence to make it clearer for the reader. With this change, the reviewer's suggestion was incorporated.

[Comment 39] L251: genus should not be abbreviated the first time its cited

**Author’s Response:** At the beginning of the results (Line 179), we already called Table S2, which is in the supplementary, so here we use the abbreviation as it is not the first time it appears. Here we think the paragraph is too long, but if the reviewer thinks it's better we can change it without any problems.

[Comment 40] L282: Maybe "Research shows" would be better

**Author’s Response:** Done.

[Comment 41] L283-285: I think this should be rephrased as follows "Furthermore, these species, often display a more generalist or tolerant behavior towards diverse environmental conditions, which may contribute to their reduced vulnerability"

**Author’s Response:** Thank you so much. Done.

[Comment 42] L286: maybe "resulting in a broader distribution range" would be better

**Author’s Response:** Thank you so much. Done.

[Comment 43] L287: As these two families represent the highest diversity within the order, using them as the sole example may appear exaggerated. There are numerous instances of restricted distribution within both families. I would recommend considering a selection of species from each family or, at the very least, a genus, to better illustrate the point.

**Author’s Response:** Thank you. We cite examples throughout the paragraph.

Line 252-258: In our study the families Libellulidae and Coenagrionidae, are more abundant and both are diverse families at a continental scale which, which may explain their wide distribution [53–54]. **Some species of Coenagrionidae are associated to degraded environments in previous study, within this family *E. metallica*** (**associated with streams with** environmental change Faria et al. 2021) **and *N. luzmarina* had high niche breadth, and Libellulidade** such as *E. fusca,* and *O. walkeri* **are associated with streams with** environmental change **environmental change** [55–56]. **In addition, *M. cupraea, H. silvarum* and *M.* *aenea* also presented greater niche** breadth..

[Comment 44] L289: Which ones? I recommend to improve this paragraph.

**Author’s Response:** Thank you so much. Done. Line 276: Ecofisiological traits as body size and thorax temperature.[Comment 45] L293: change ‘and’ by ‘, whereas’

**Author’s Response:** Thank you so much. Done.

[Comment 46] L295: Nonetheless]

**Author’s Response:** Thank you for the comment We tried to modify some paragraphs and lot of sentences was removed.

[Comment 47] L297: Do the data presented in this work support this statement? If so it would be interesting to include here.

**Author’s Response:** As the GLM result (Table 4) demonstrates a positive relationship between NB and abundance, we can support this result, at least for our data. Theory predicts that species with higher NB values are generalists and live in environments with a wider range of environmental conditions (Doledec 2000 and 2017).

[Comment 48] L298: The analysis shows that..

**Author’s Response:** Thank you so much. Done

[Comment 49] L306-308: This paragraph needs to be rewritten

**Author’s Response:** Thank you. We agree with the notes, and therefore, we have rewritten a lot of topics of discussion. Please, see it seems better.

[Comment 50] L308-312: This paragraph should be revised according to the changes in the previous statement.

**Author’s Response:** Thank you. We agree with the notes, and therefore, we have rewritten a lot of topics of discussion. Please, see it seems better.

[Comment 51] L309: Heino & Grönroos [2]

**Author’s Response:** Thank you. we have rewritten a lot of topics of discussion, this reference has been removed from this place.

[Comment 52] L313: Ribeiro et al. [49]

**Author’s Response:** Thank you. we have rewritten a lot of topics of discussion, this reference has been removed.

[Comment 53] L315: Should be another paragraph

**Author’s Response:** Thank you. We agree with the notes, and therefore, we have rewritten a lot of topics of discussion. Please, see it seems better.

[Comment 54] L332: I recommend to use an species of Zygoptera as an example

**Author’s Response:** Thank you. we have rewritten a lot of topics of discussion, this has been removed.

[Comment 55] L344: May [15]

**Author’s Response:** Thank you. Done.

[Comment 56] Fig 4. The abbreviation of the genus could be the first letter (as used traditionally). I don't think there is a need to use this type of abbreviation (its not used in the ms for example). If its use is justified I would at least add an space between genus abbreviation and specific epithet.

**Author’s Response:** Thank you, done. We use at Fig. 4.

[Comment 57] Fig. 2 A map of brazil or South America with the state highlighted would be useful for reference.

**Author’s Response:** Thank you. Done.

[Comment 58] Supporting Information: The SD is confusing in the table, it should be separated by color or bars to clearly differentiate between species. Also the Table description should be improved. The c in the temperature should be capital "C".

**Author’s Response:** Thank you. Done. SD stands for standard deviation of thoracic and temperature averages. The species without SD have only 1 individual and it was not possible to calculate. Again, thank you for colors suggestion, this is much better. It was really very confused and with lot of mistakes, sorry.

References

Castillo-Pérez EU, Suárez-Tovar CM, González-Tokman D, Schondube JE, Córdoba-Aguilar A. Insect thermal limits in warm and perturbed habitats: Dragonflies and damselflies as study cases. J Therm Biol. 2022; 103: 103164. https://doi.org/10.1016/j.jtherbio.2021.103164
